# Populations of aspen (*Populus tremuloides* Michx.) with different evolutionary histories differ in their climate occupancy

**DOI:** 10.1002/ece3.2102

**Published:** 2016-03-30

**Authors:** Burke T. Greer, Christopher Still, Glenn T. Howe, Christina Tague, Dar A. Roberts

**Affiliations:** ^1^ Forest Ecosystems and SocietyOregon State University321 Richardson HallCorvallisOregon97331; ^2^ Department of GeographyUniversity of California Santa Barbara1832 Ellison HallSanta BarbaraCalifornia93106; ^3^ Bren School of Environmental Science and ManagementUniversity of California2400 Bren HallSanta BarbaraCalifornia93106‐5131

**Keywords:** Biodiversity, biogeography, climate niche, ecological genetics, phyloclimatic modeling

## Abstract

Quaking aspens (*Populus tremuloides* Michx.) are found in diverse habitats throughout North America. While the biogeography of aspens' distribution has been documented, the drivers of the phenotypic diversity of aspen are still being explored. In our study, we examined differences in climate between northern and southwestern populations of aspen, finding large‐scale differences between the populations. Our results suggest that northern and southwestern populations live in distinct climates and support the inclusion of genetic and phenotypic data with species distribution modeling for predicting aspens' distribution.

## Introduction

Quaking aspen (*Populus tremuloides* Michx.) thrives in a variety of landscapes across North America. In the Intermountain West and Rocky Mountains, aspens are found in dense groves with spruce and fir at middle elevations, and in pure stands or in isolated groves at tree line (Shepperd et al. 2000). At its lowest elevations in Nevada and Utah (150–300 m), aspens are found in riparian corridors (Mueggler 1988). In the Sierra Nevada Mountains of California, aspens are found in riparian corridors, on slopes, and in isolated pockets and krümmholz stands (Shepperd et al. 2006). In contrast, North America's eastern and northern aspens are found most often at swamp and stream margins (Barnes and Wagner 2002). In the southwest region of North America, aspens occur on sites typically fed by snowmelt, whereas in the north and east, they rely on winter snowpack and summer rains (Barnes and Wagner 2002). Unlike aspens in the southwest, northern aspens are only able to colonize upland sites when canopy openings become available following fire or other disturbances, and on these sites, they are later outcompeted by other species (Barnes and Wagner 2002).

Aspen stands are made up of one or multiple clones which propagate through sexual reproduction using windblown pollen and seeds, and asexual reproduction through clonal growth from root suckering (Barnes 1975; Barnes and Wagner 2002; Mock et al. 2008). Although the Rocky Mountains are famous for their large clonal stands of aspen, large clonal stands are absent from the Great Lakes region and Central Canada (Mitton and Grant 1996). These differences suggest that asexual reproduction is much more common in the southwestern portion of aspens' range (Kemperman and Barnes 1976) and that sexual reproduction occurs during short “windows of opportunity” (Jelinski and Cheliak 1992). Nonetheless, throughout its range, the sizes and distributions of aspen clones depend on the combined success of sexual and asexual reproduction (Mock et al. 2008).

Species distributions are constrained by evolutionary history, the ability and opportunity to disperse into new environments, the amount of adaptive phenotypic variation (including adaptation to climate), and gene flow among the extant populations (Kirkpatrick and Barton 1997). Analyses of neutral genetic markers suggest that aspens' current distribution has been weakly constrained by evolutionary history, while gene flow has been extensive. For example, analysis of range‐wide genetic diversity using SSR markers suggests that diploid aspens are weakly differentiated within the northern and southwestern portions of the species' range (Callahan et al. (2013). These “northern” and “southwestern” clusters are roughly separated by a boundary consisting of the maximum extent of the Pleistocene glaciation and the continental divide (see fig. 1 in Callahan et al. 2013). Although the northern cluster was found to have greater genetic diversity, it had no strong geographical structure. This suggests that gene flow is high and/or the northern cluster resulted from a cohesive northward migration of populations during the retreat of the glaciers. The southwestern cluster is very different, having lower genetic diversity and greater geographical structure. The authors hypothesized that the southwestern cluster consists of “stable edge” populations – instead of moving northward, these populations seem to have migrated up and down the mountains and hillslopes tracking changes in climate. Additionally, Callahan et al. (2013) speculated that the northern and southwestern clusters are adapted to different climates because the northern cluster inhabits a mesic, continental climate, whereas the southwestern cluster inhabits a climate that is semiarid.

Empirical information on aspens' adaptation to climate comes from analyses of climate envelopes and common garden studies. Range‐wide climate envelopes have been characterized by Rehfeldt et al. (2009) and Worrall et al. (2013). Rehfeldt et al. (2009) found that aspens' distribution was primarily driven by three climate variables: an annual dryness index, the ratio of summer to annual precipitation, and an index incorporating growing season precipitation and growing degree‐days. Using an updated version of this model, Worrall et al. (2013) found that maximum summer temperatures and summer precipitation (April–September) were the best predictors of aspens' range‐wide distribution.

Traits associated with local climatic adaptation of aspen populations have been identified using common garden studies. For example, in a reciprocal transplant study in Alberta, Canada, tree diameter and height were strongly related to the latitudes from which each population originated (Gylander et al. 2012). In another reciprocal transplant study of ten aspen populations from Western Canada and Minnesota, tree height, total biomass, and the timing of budbreak were strongly related to latitude (Schreiber et al. 2013), and in a related study, the timing of budbreak was associated with total growing degree‐days (Li et al. 2010). Finally, using a reciprocal transplant study and species distribution models, relationships between tree heights and climate variables were used to project the future growth of aspens for 2020, 2050, and 2080 under four Intergovernmental Panel on Climate Change (IPCC) emissions and population growth scenarios (Gray et al. 2011). However, because the study of Gray et al. (2011) was restricted to Western Canada, it did not address the larger‐scale differences between the northern and southwestern portions of aspen's range.

Given these aspen–climate relationships, and the work of Callahan et al. (2013), we decided to quantify similarities and differences between the climates of North America's northern and southwestern aspen and to characterize the dominant climatic controls on their distributions. We extended the approach of Rehfeldt et al. (2009) and Worrall et al. (2013) by creating and comparing ensemble SDMs (species distribution models) for the entire range of aspen, as well as for the northern and southwestern clusters described by Callahan et al. (2013). We also used 10 different statistical modeling methods when creating our ensemble models, as opposed to the single approach used in earlier studies.

## Methods

**Figure 1:**
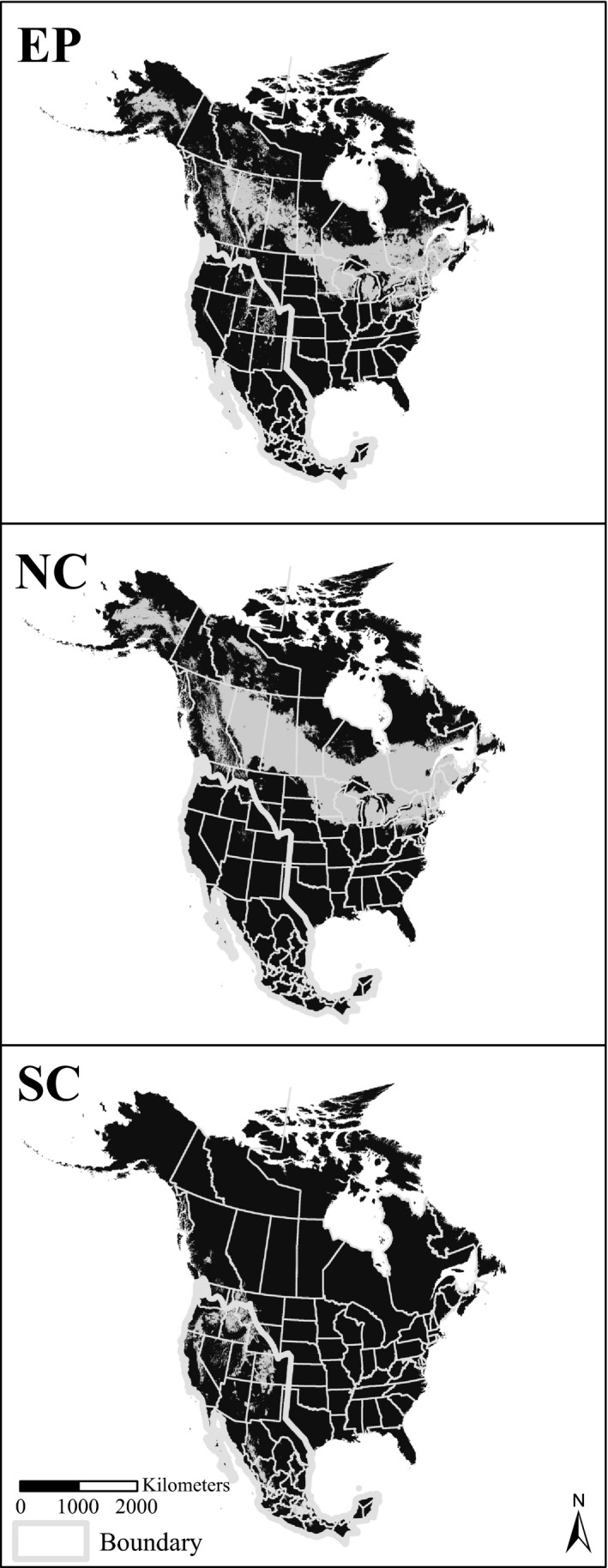
Predictive maps of aspen's distribution. The white line is the boundary for the presence data of northern cluster and southwestern cluster.

To better understand climate differences between the northern and southwestern clusters identified by Callahan et al. (2013), we used methods that have been used to measure niche overlap between SDMs (Warren et al. 2008, 2014; Franklin 2010, 2013). For these analyses, three ensemble distribution models were created: one for the EP (entire population), one for the NC (northern cluster), and one for the SC (southwestern cluster). The NC and SC range boundaries (the gray boundary in Fig. 1) are adapted from a map of the distributions of the northern and southwestern clusters defined by Callahan et al. (2013) using SSR data. After the creation of the ensemble models, the predicted distributions and climates were compared between EP, NC, and SC.

We used an ensemble modeling approach because it provides more robust estimates of distributions than are possible when using a single model (Araujo and New 2007). Models were built with the biomod2 package (Thuiller et al. 2014) in R (R Core Team, 2014) using a high‐performance computing cluster at the College of Forestry at Oregon State University. Each final predicted range, and climate association was calculated from 200 models derived from 20 runs of 10 model types (Tables 1 and 2, Appendix S4). The final models are the averages of all models that met a minimum accuracy of 0.6 measured using the TSS (true skill statistic) (Allouche et al. 2006). A value of 1 for TSS represents perfect correspondence between predicted and measured species presences and absences, whereas a value of 0 represents no correspondence. The input presence and absence data (see Appendix S1) were from Worrall et al. (2013), and the climate data (Table 2, Appendix S1) were either taken directly or calculated from the WorldClim dataset (Hijmans et al. 2005). For EP, we used an equal number of the presence and absence points, for a total of ~100,000 points. For NC and SC, aspen presences within each region were used in conjunction with all absences across the range of aspen (roughly 94,000 total points for NC and 53,000 total points for SC, see Table 3). We used 80% of the input points for model training and withheld 20% for model testing.

**Table 1:** Modeling methods

GLM: Generalized linear model
GAM: Generalized additive model
GBM: Generalized boosting model (also known as boosted regression tree)
CTA: Classification tree analysis
ANN: Artificial neural network
SRE: Surface range envelope (BIOCLIM)
FDA: Flexible discrimination analysis
MARS: Multiple adaptive regression splines
RF: Random forests
MAXENT: Maximum entropy

**Table 2:** Climate layers used for modeling

Growing degree‐days (dd5), unitless
Mean annual precipitation (MAP), mm/year
Potential evapotranspiration (PET), mm/year
PET/MAP, unitless
AET/MAP, unitless
Precipitation seasonality (summer precipitation/winter precipitation, or psea), unitless
Temperature maximum (tmaxyr), °C
Temperature minimum (tminyr), °C
Temperature range (trang), °C

**Table 3:** Presence and absence data density

	Entire population	Southwestern cluster	Northern cluster
Total points	97,486	51,825	94,968
Presence points	48,179	2518	45,661
Absence points	49,307	49,307	49,307
Area bounding presence points (km^2^)	21,252,207	4,801,382	16,450,825
Total density for area bounding presence points (pts/km^2^)	0.0046	0.0108	0.0058
Presence density per area bounding presence points	0.0023	0.0005	0.0028
Absence density per area bounding presence points	0.0023	0.0103	0.003

Schoener's statistic and the modified Hellinger's statistic, *D* and *I* were used to evaluate niche overlap (Schoener 1968; Warren et al. 2008). The *D* and *I* metrics are defined as follows:

(1) $$D\left(p_{x.i},\;p_{y.i}\right)=1-\frac{1}{2}\sum \left|p_{x.i}-p_{y.i}\right|,$$

(2) $$I\left(p_{x.i},\;p_{y.i}\right)=1-\frac{1}{2}\sqrt{\sum {\left(\sqrt{p_{x.i}}-\sqrt{p_{y.i}}\right)}^{2}},$$

where *p*_*x.i*_ and *p*_*y.i*_ are the probabilities of occurrence for species *x* and *y* at location *i*. For *D* and *I*, a value of zero indicates no niche overlap, and a value of 1 indicates complete niche overlap.

The relative contributions of each climate variable to each model are also important for understanding why NC, SC, and EP may differ. This was measured by biomod2 using the following methodology. Once a model was trained and run, the model was run a second time with the values of one of the input variables randomized. One minus the correlation between the original prediction (with all the original variables) and the new prediction (with the randomized variable) provided an index of relative importance of the climate variable. A higher relative importance value for a given climate variable indicates greater influence on the modeled species distribution.

## Results

Maps of predicted distributions for the ensemble models EP, NC, and SC are shown in Figure 1. The TSS values (i.e., model evaluation metrics) for these three models were 0.868, 0.727, and 0.915, respectively, with all scores indicating a good model fit to the presence and absence data. EP predicted contiguous aspen habitat in Central Canada, the Great Lakes region, the northern Rocky Mountains, pockets in Utah and Colorado, the Sierra Nevada Mountains of California, and isolated areas in Arizona, New Mexico, and Mexico. These predictions generally agreed with Little's aspen range maps (Little 1971) and the predicted distributions described by Rehfeldt et al. (2009) and Worrall et al. (2013). However, the predicted distributions for NC and SC were very different and suggested that NC and SC aspens occupy different climates.

To further test the hypothesis that the climate envelopes of NC, SC, and EP were different, we compared *D* and *I* among the models. *D* and *I* were very low between NC and SC (0.006 and 0.018, respectively), showing that the predicted climate occupancy was different between the northern and southwestern clusters. *D* and *I* were also very low between SC and EP (0.037 and 0.089), but *D* and *I* between EP and NC were much higher (0.710 and 0.820). These results suggested that SC inhabits a different climate than EP or NC and that this climate was closer to the edge of aspens' overall climate niche compared to NC.

**Figure 2:**
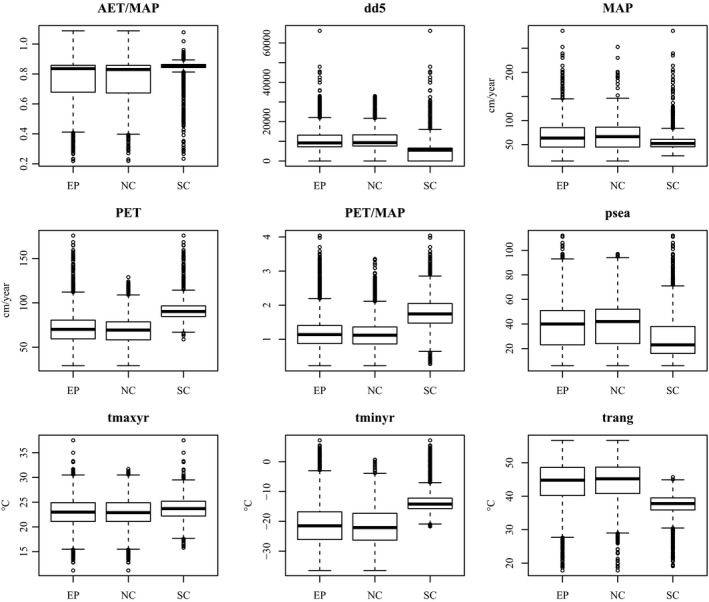
Boxplots of climate variables at the aspen presence locations for entire population, northern cluster, and southwestern cluster. Axes without labels are unitless.

Because NC and SC were created from different aspen presence datasets, we examined how the climate datasets differed between EP, NC, and SC. Boxplots of the climate variables at the aspen presence points used in each ensemble model are shown in Figure 2. The median and interquartile range of SC compared to EP and NC showed that SC had very different AET/MAP, growing degree‐days (dd5), PET, PET/MAP, precipitation seasonality (psea), temperature minimum (tminyr), and temperature range (trang). The boxplots of the climate variables for EP and NC were more similar. Wilcoxon's rank‐sum tests also showed that the climate variables for SC were statistically different from both EP and NC (*P* < 0.0001). Also, Wilcoxon's rank‐sum tests showed that the climate variables for EP and NC were also statistically different from each other (*P* < 0.01679). Finally, the climatic ranges of the absence data (which were the same for EP, NC, and SC) extended beyond the ranges of the presence data for EP, NC, and SC (Appendix S3).

**Figure 3:**
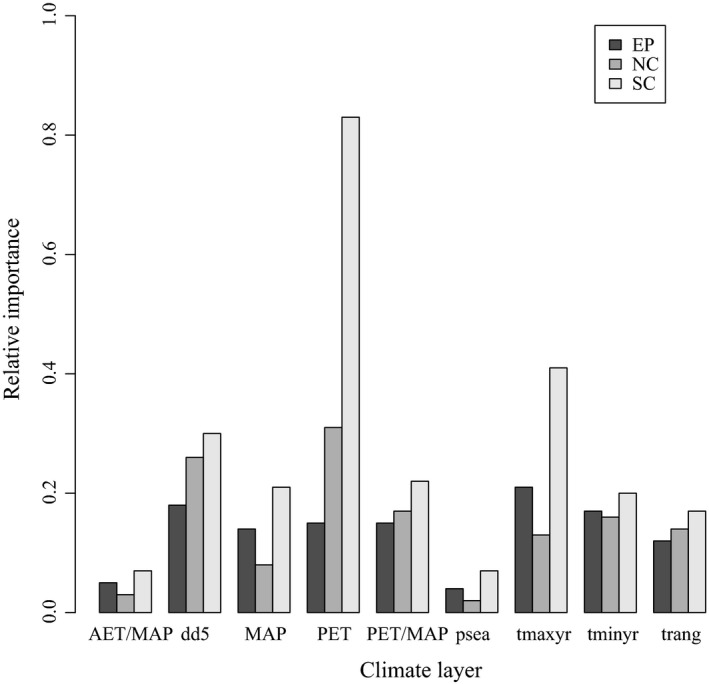
The relative importance of each climate layer. Only model runs where true skill statistic was >0.6 were included in the final ensemble model, and each model method varied in the variables important to each model; therefore, the variable importance reported is weighted by the actual model contributions to each ensemble model (entire population, northern cluster, and southwestern cluster, see Appendix S4 for tabular data).

The relative importance data (Fig. 3) showed that EP, NC, and SC had climate variables that were similar in importance, ranging between ~0.15 and ~0.35. The biggest exceptions were PET and temperature maximum. PET was much more important in SC (0.83) than in either EP (0.15) or NC (0.31), and temperature maximum was more important in SC (0.41) than in NC (0.13) or EP (0.21). The importance of growing degree‐days was similar between NC (0.26) and SC (0.30), but less important in EP (0.18). Mean annual precipitation was less important in NC (0.08) than in either EP (0.14) or SC (0.21). The boxplots and Wilcoxon's rank‐sum tests showed differences in PET, temperature maximum, growing degree‐days, and mean annual precipitation between SC and NC. PET and temperature maximum were greater in SC than in NC, and growing degree‐days and mean annual precipitation were lower in SC than in NC (Fig. 2).

Overall, the comparisons of the ensemble models' distributions and climates, the comparisons of *D* and *I*, and the relative importance of each climate variable, all suggested that the northern and southwestern clusters occupy different climates.

## Discussion

Our results show that aspens in the northern and southwestern clusters occupy different climates. This is indicated by the large differences in predicted distributions between NC and SC, the small amount of climate overlap (low *D* and *I*), the differences in the climates of the aspen presence points for SC relative to NC and EP, and the dissimilarity in the relative importance of the climate variables to each model. Furthermore, the relative importance of the climate variables showed that PET and growing degree‐days were more important in NC and SC than in EP. In contrast, mean annual precipitation and temperature maximum were more important in SC and EP than in NC. If aspen populations in the EP, NC, and SC clusters responded similarly to climate, we would expect the relative importance of the climate variables to the models to remain the same. Instead, the relative importance of the climate variables differs.

Our overall conclusions are also supported by finer‐scale studies documenting population differences in adaptive traits that are associated with latitude and climate variables (Li et al. 2010; Gylander et al. 2012; Schreiber et al. 2013). In addition, Gray et al. (2011) found associations between growth performance and climate envelopes in Western Canada, while Mock et al. (2012) found a potential relationship between the frequency of aspen triploidy and an ombrothermic index. Also, our finding that SC occupies a different climate than EP, or NC, is consistent with the hypothesis of Worrall et al. (2013) that aspens in the Southwestern United States live on the edge of aspens' overall climate niche.

Our findings rest on three assumptions: The first assumption is that the differences between NC and SC do not result from spatial autocorrelation alone. For example, the spatial clustering of similar values in climate layers relative to the input data presence and absence points could introduce bias to SDM predictions. Secondly, we assume that differences between the ensemble model results represent biological differences between the clusters and not differences between the model methods that comprise each ensemble (see Appendix S4). However, even if the contributing model methods differ between the ensemble models, we used robust techniques to measure our models' predictive powers and found that TSS was high in each final ensemble model. The final assumption is that the climate occupancy we measured reflects the climatic tolerance or adaptive potential of each cluster. This assumption is well supported by results from regional common garden studies (Gylander et al. 2012; Schreiber et al. 2013). Broader common garden studies could be used to further test this hypothesis, but common garden studies have their limitations. Common garden studies do not measure fitness per se, but, instead, typically use short‐term growth as a surrogate. Furthermore, because they usually use planted seedlings, they do not capture climatic tolerances of many important parts of the life cycle – for example, flowering, pollination, fertilization, seed production, germination, and seedling establishment or clonal reproduction. Thus, in many respects, SDMs are probably better than common garden experiments for inferring climatic tolerances of naturally regenerated forests. Another advantage of using SDM methods to infer climatic tolerances is that it is possible to study more populations from a greater geographical area than that is possible using common garden tests. For example, we analyzed 97,486 presence and absence locations. Therefore, using SDMs, we widened the analysis of aspen–climate comparisons.

The regional differences in our SDMs, as well as results from common garden studies from aspen and other forest tree species, strongly suggest that NC and SC differ because of evolutionary adaptation to alternative climates. However, other explanations are possible. First, the results of Callahan et al. (2013) suggest that the differences we observed may have resulted from geographically distinct, climate‐independent, evolutionary pathways. That is, these differences may have been largely random, or driven by selection to climates in the distant past, rather than the recent climates we measured. Second, the geographical differences we observe may be driven by differences in biotic interactions between the regions, rather than differences in adaptation to climate alone (e.g., interspecific competition or pathogen interactions). However, despite these alternative explanations, it is doubtful that any of these possibilities explains our observations independent of climate impacts.

Global climate change is expected to transform the distributions of many forest trees (Noss 2001), and regional or local differences in climatic adaptation are important to consider when predicting the impacts of climate change and designing effective adaptation and mitigation strategies. Our findings strongly suggest that aspen distribution models should be tailored to reflect local adaptation to climate. By doing so, we should improve projections of future aspen distributions (or at least potential habitat) and, thus, improve the success of assisted migration. Future range‐wide studies in aspen that merge genetic and finer‐scale SDM approaches will help scientists and natural resource managers to understand species–environment interactions, which will lead to better aspen forest management.

## Conflict of Interest

None declared.

## Supporting information


**Appendix S1.** Data sources.
**Appendix S2.** Methods for climate data layer creation.
**Appendix S3.** Absence data boxplot.
**Appendix S4.** Ensemble model methods.
