# Supplementary material for: Populations of aspen (Populus tremuloides Michx.) with different evolutionary histories differ in their climate occupancy
Source: Ecol Evol. 2016 Mar 30;6(9):3032–9. doi: 10.1002/ece3.2102 (PMC4863026; doi:10.1002/ece3.2102)
Supplement: Supplementary file 1 — Appendix S1. Data sources. Appendix S2. Methods for climate data layer creation. Appendix S3. Absence data boxplot. Appendix S4. Ensemble model methods. [file ECE3-6-3032-s001.docx]

# Supplemental:

**Appendix S1: Data sources**

The raw presence-absence data is the same dataset used by Worrall et al. (2013). Supplement 1 of Worrall et al. (2013) notes that the combined presence-absence dataset is obtained from inventory sample plots, ecology plots, and herbarium accessions. Of special note, there are some aspen locations whose presence may be confused with other species: for example, the Maritime Provinces of Canada do not distinguish between bigtooth aspen (*Populus grandidentata*) and trembling aspen.

**Appendix S2: Methods for Climate Data Layer Creation**

**CLIMATE DATA**
Climate layers (Table 1.) were either used directly or calculated from datasets provided by WorldClim (Hijmans, et al., 2005). The spatial resolution for all data sources is thirty arc-seconds, or approximately 800 meters. This resolution, while coarser than the presence/absence data, is the finest scale of analysis for the models.

The specifics of the climate layers are as follows:
The Growing Degree Days metric represents the sum of monthly growing degree days (GDD) over 5°C for the year. GDD represents cumulative warmth for the year and is an index for the warmth of a site above a threshold degree of 5°C, which is used as a minimum threshold above which plants are most productive. This calculation is taken from Kira (1976), who originally referred to it as a ‘warmth index.'

Monthly GDD are created using the following scenarios using temperature maxima and minima adapted from Kira’s warmth index (Kira, 1991):

- 1. When the minimum temperature of a month is above 5°C, all days in that month are assumed to be above temperature 5°C. Thus, GDD of the month is calculated as:

- 1. If the maximum temperature of a month is below 5°C, all days in that month are assumed to be below 5°C and the GDD of the month is 0:

- 1. If the maximum and minimum temperatures span the 5°C threshold, then the number of days above 5°C was calculated using linear interpolation. When Tmax and Tmin are an equal magnitude from the 5°C threshold (e.g., 7.5°C and 2.5°C), this ratio would be 0.5; relative to 0.5, this ratio would increase (decrease) if Tmax (Tmin) were farther away from 5°C than Tmin (Tmax). So Month Growing Degrees would be ((Tmax + 5°C)/2 - 5°C).

Mean Annual Precipitation (MAP) represents the mean total yearly precipitation.

Potential evapotranspiration represents the total PET in mm per year. The data source is WorldClim (Hijmans et al., 2005) where PET is calculated using the Hargreaves et al. (1985) model. Mean monthly temperature (Tmean), mean monthly temperature range (TD) and mean monthly extra-terrestrial radiation (RA, at top of atmosphere) were used to calculate mean PET for a raster of the world using this formula:

$$PET=0.0023*RA*(Tmean+17.8)*TD^{0.5}$$

These data are summarized for the 1950 to 2000 period using available rasterized data available from WorldClim databases and extra-terrestrial radiation calculated using Allen et al’s method (1998), where the solar constant, solar declination and the time of the year are used to determine RA. This RA value is dependent on latitude.

AET/MAP is actual evapotranspiration divided by MAP and is supplied by WorldClim.

PET/MAP is PET divided by MAP.

Precipitation seasonality represents the ratio of winter precipitation to summer precipitation. It is calculated using ArcGIS from monthly precipitation total summaries provided by Worldclim. Winter precipitation is represented by adding monthly precipitation totals for December, January, February and March. Summer precipitation is represented by adding monthly precipitation totals for June, July, August and September. The winter total is then divided by the summer total to produce Precip Seasonality.

Temperature Maximum and temperature minimum are the mean monthly maximum and minimum temperatures across the year.

TRNG represents the range of temperatures. It is calculated from the yearly average maximum (Tmax) and minimum (Tmin) data layers. It is also referred to as Trange_m14 in the MaxEnt outputs.

$$TRNG=T\max-T\min$$

**Appendix S3: Absence Data Boxplots**
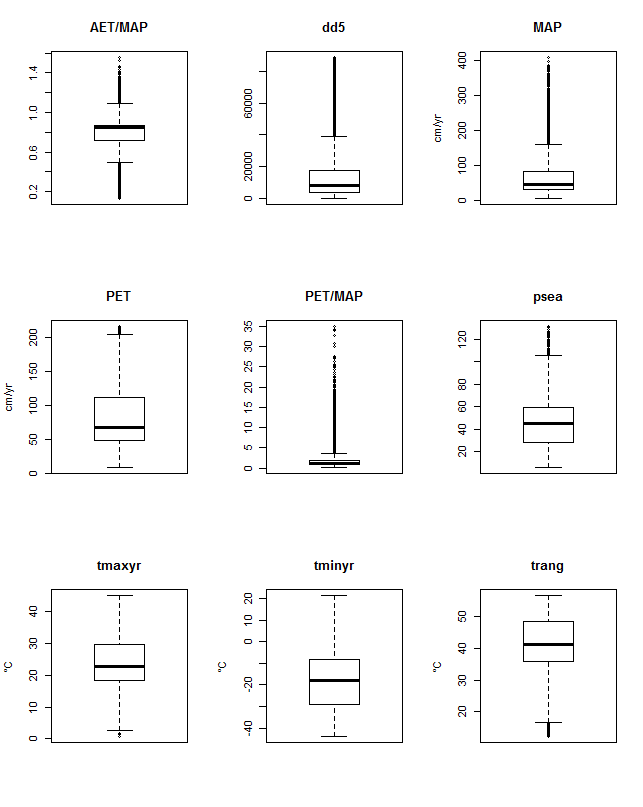

Figure 1. Boxplots of the absence data used in EP, NC and SC.

**Appendix S4: Ensemble model methods**

Table 1. Models included in ensemble (TSS>.6) listed by model type.

EP NC SC

GBM 0 0 20

GLM 5 20 20

GAM 0 16 20

CTA 6 20 20

ANN 0 0 20

SRE 0 0 20

FDA 0 0 20

MARS 0 3 20

RF 20 20 20

MAXENT 12 20 20

Table 2. Average variable importance measured across all methods

EP NC SC

AET/MAP 0.06 0.06 0.07

dd5 0.22 0.27 0.30

map 0.21 0.19 0.21

pet 0.25 0.44 0.83

PET/MAP 0.22 0.22 0.22

psea 0.04 0.03 0.07

tmaxyr 0.33 0.16 0.41

tminyr 0.29 0.24 0.20

trang 0.20 0.17 0.17

Table 3. Weighted average variable importance across all models

Weighted Variable Importance EP NC SC

AET/MAP 0.05 0.03 0.07

dd5 0.18 0.26 0.30

map 0.14 0.08 0.21

pet 0.15 0.31 0.83

PET/MAP 0.15 0.17 0.22

psea 0.04 0.02 0.07

tmaxyr 0.21 0.13 0.41

tminyr 0.17 0.16 0.20

trang 0.12 0.14 0.17

Kira, T. (1991). Forest Ecosystems of East and Southeast-Asica in a Global Perspective. *Ecological Research, 6*(2), 185-200. doi: 10.1007/bf02347161

Worrall, J. J., Rehfeldt, G. E., Hamann, A., Hogg, E. H., Marchetti, S. B., Michaelian, M., & Gray, L. K. (2013). Recent declines of Populus tremuloides in North America linked to climate. *Forest Ecology and Management, 299*, 35-51. doi: DOI 10.1016/j.foreco.2012.12.033
